# The effect of heat acclimation or acclimatisation on physiological markers of heat adaptation: preliminary meta-analysis data

**DOI:** 10.1186/2046-7648-4-S1-A110

**Published:** 2015-09-14

**Authors:** Christopher J Tyler, Tom Reeve, Gary J Hodges, Stephen S Cheung

**Affiliations:** 1 Department of Sport and Exercise Science, University of Roehampton, London, UK; 2 Environmental Ergonomics Laboratory, Department of Kinesiology, Brock University, St. Catharines, Canada

## Introduction

Exercise in the heat places a greater physiological strain upon the body than exercising in temperate conditions, so a number of strategies have been adopted to attenuate this strain. Heat acclimation (or acclimatisation) (HA) has regularly been reported to induce beneficial cardiovascular and thermoregulatory adaptations. However, the magnitudes of benefit reported range from none to substantial, and the differences reported may be due to a wide range of HA protocols being used. The aim of this meta-analysis was to quantify the magnitude of effect that HA has on key physiological markers of adaptation, and to see whether the magnitude of effect is related to the volume or intensity of heat stress experienced.

## Methods

The PubMed database was searched (09/01/15) using the first-order search terms *acclimation, acclimatization, acclimatisation *and *adaptation *and second-order search terms *heat, exercise, performance, capacity *and *training*. Using the four-stage process identified in the PRISMA statement the initial number of results (9,369) was reduced to 92. Data (N, mean, SD) were extracted from these articles in duplicate or triplicate. A subset of the data (n = 46 manuscripts) is presented here; manuscripts were included if resting core temperature (T_core_), resting heart rate (HR), resting plasma volume (PV) and/or core temperature at sweat onset (T_sweat onset_) data were reported. All HA protocols regardless of duration, frequency, ambient conditions or exercise modality were used. Hedge's *g *( ± 95% CI) were calculated and Spearman's correlation analyses were performed between the effect size and total HA time (HA_time_), and HA temperature (HA_temp_).

## Results

The 46 manuscripts reviewed used a mean (SD) of 9 (0) [range: 4 - 16] HA sessions separated by 0 (0) [0 - 1.5] days. Total HA_time _was 868 (558) min [150 - 2,880], and the HA_temp _and HA_humidity _were 39 (5) °C [28 - 50] and 36 (16) % [14 - 86], respectively.

## Conclusion

HA is an effective way to reduce resting T_core _and HR; increase resting PV, and lower the T_sweat onset_. The magnitude of effect appears to be independent of HA_time _or HA_temp _for each of the 4 variables with the exception of T_sweat onset_, which may be inversely related to HA_time_; however, these latter data are derived from only 6 investigations.

**Table 1:** The effect of HA on resting T_core_, resting HR, resting PV and T_sweat onse__t_.

	Articles	Groups	N	Hedges *g *(95% CI)	Mean Δ	HA_time_	HA_temp_
Resting T_core_	35	40	372	-0.62 (-0.77, -0.47)	-0.17 ± 0.13 °C	*r *= -0.01^NS^	*r *= 0.02^NS^

Resting HR	21	27	247	-0.60 (-0.78, -0.41)	-5 ± 4 bpm	*r *= -0.20^NS^	*r *= -0.13^NS^

Resting PV	17	18	183	+0.57 (0.36, 0.79)	+3.5 ± 3.6 %	*r *= -0.37^NS^	*r *= -0.20^NS^

T_sweat onset_	6	9	85	-0.88 (-1.17, -0.59)	-0.24 ± 1.3 °C	*r *= -0.83**	*r *= -0.29^NS^

^** ^= P <0.01; ^NS ^= p = 0.07 - 0.50

